# A New Kid on the Block: Sacituzumab Govitecan for the Treatment of Breast Cancer and Other Solid Tumors

**DOI:** 10.3390/molecules26237294

**Published:** 2021-12-01

**Authors:** Giuliana Pavone, Lucia Motta, Federica Martorana, Gianmarco Motta, Paolo Vigneri

**Affiliations:** 1 Center of Experimental Oncology and Hematology, A.O.U. Policlinico “G.Rodolico-S.Marco”, 95123 Catania, Italy; giulianapavone91@gmail.com (G.P.); fede.marto.fm@gmail.com (F.M.); gianmarcomotta90@gmail.com (G.M.); vigneripaolo@gmail.com (P.V.); 2 Medical Oncology Unit, A.O.U. Policlinico “G.Rodolico-S.Marco”, 95123 Catania, Italy; 3 Department of Clinical and Experimental Medicine, University of Catania, 95123 Catania, Italy

**Keywords:** antibody-drug conjugates, Sacituzumab govitecan, Trop-2, breast cancer

## Abstract

Human trophoblast cell-surface antigen-2 (Trop-2) is a membrane glycoprotein involved in cell proliferation and motility, frequently overexpressed in epithelial tumors. Thus, it represents an attractive target for anticancer therapies. Sacituzumab govitecan (SG) is a third-generation antibody-drug conjugate, consisting of an anti-Trop-2 monoclonal antibody (hRS7), a hydrolyzable linker, and a cytotoxin (SN38), which inhibits topoisomerase 1. Specific pharmacological features, such as the high antibody to payload ratio, the ultra-toxic nature of SN38, and the capacity to kill surrounding tumor cells (the bystander effect), make SG a very promising drug for cancer treatment. Indeed, unprecedented results have been observed with SG in patients with heavily pretreated advanced triple-negative breast cancer and urothelial carcinomas, and the drug has already received approval for these indications. These results are coupled with a manageable toxicity profile, with neutropenia and diarrhea as the most frequent adverse events, mainly of grades 1–2. While several trials are exploring SG activity in different tumor types and settings, potential biomarkers of response are under investigation. Among these, Trop-2 overexpression and the presence of *BRCA1/2* mutations seem to be the most promising. We review the available literature concerning SG, with a focus on its toxicity spectrum and possible biomarkers of its response.

## 1. Introduction

**Figure 1:**
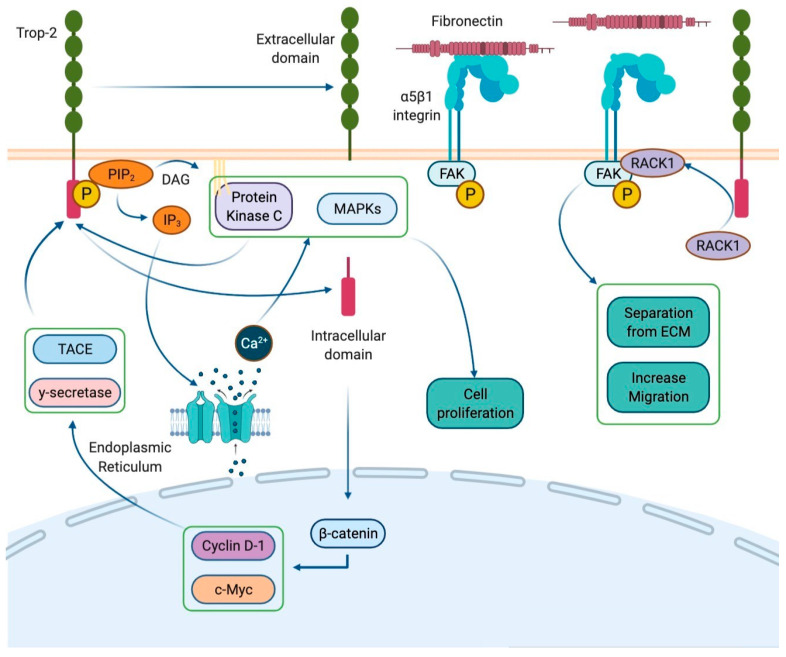
Trop-2 signal transduction. Trop-2 is a membrane receptor consisting of an extracellular domain, a transmembrane domain, and an intracellular domain. Its cytoplasmatic domain contains a phosphatidyl-inositol 4,5-bisphosphate (PIP2) binding site. The interaction with PIP2 allows the phosphorylation of the receptor by protein kinase C (PKC). This determines the cleavage of PIP2 into inositol 1,4,5-triphosphate (IP3) and diacylglycerol (DAG) by phospholipase C. IP3 remains in the cytoplasm and mediates the accumulation of intracellular calcium by opening the calcium channels located on the endoplasmic reticulum. Trop-2-induced calcium release leads the recruitment of the mitogen-activated protein kinases (MAPKs), which promote cell proliferation. Furthermore, Trop-2 undergoes the cleavage into two parts by the proteases γ-secretase and TNF-α converting enzyme (TACE). The intracellular domain moves to the nucleus and colocalizes with a β-catenin resulting in the up-regulation of Cyclin D1, which fosters the cell cycle progression. Finally, Trop-2 would seem to be involved in the loss of cell–substrate adhesion, i.e., separation from the extracellular membrane (ECM), and in the promotion of cell migration due the activation of the β1–integrin–RACK1–FAK–Src signaling axis.

Human trophoblast cell-surface antigen-2 (Trop-2), also known as tumor-associated calcium signal transducer 2 (TACSTD2), is a membrane receptor encoded by the gene *TACSTD2* located on chromosome 1p32. This cell-surface glycoprotein, originally identified in human trophoblastic tissue, is commonly expressed in a variety of normal and neoplastic epithelial cells [1]. In physiological conditions, Trop-2 acts as a calcium signal transducer with a cytoplasmatic domain which contains a phosphatidyl-inositol 4,5-bisphosphate (PIP2) binding site. When PIP2 binds to Trop-2, it undergoes phosphorylation by protein kinase C (PKC). This interaction induces PIP2 cleavage into inositol 1,4,5-triphosphate (IP3) and diacylglycerol (DAG) by phospholipase C. In the cytoplasm, IP3 mediates calcium accumulation by opening specific channels in the endoplasmic reticulum. Calcium release recruits mitogen-activated protein kinases (MAPKs) such as ERK1/2, stimulating cell proliferation [2]. Furthermore, the Trop-2 intra-cytoplasmatic domain undergoes cleavage and translocates in the nucleus, where it colocalizes with β-catenin and up-regulates Cyclin D1 and c-Myc expression, promoting cell cycle progression [3,4] (Figure 1).

In cancer cells, Trop-2 overexpression stimulates growth and metastatic potential by promoting cell proliferation and migration. This protein is also involved in the epithelial-to-mesenchymal transition (EMT) since it determines E-cadherin down-regulation and vimentin expression, resulting in cell migration and stem cell-like properties [5,6,7,8]. Additionally, Trop-2 enhances cellular motility and invasion through the up-regulation of integrin-dependent signaling [3,4]. Although Trop-2 overexpression seems to be related to enhanced tumor aggressiveness and inferior prognosis, it can also be exploited as a target for anticancer therapies [9,10,11,12,13]. In the last decade, many in vitro, ex vivo, and in vivo evaluations posed the bases for Trop-2-directed therapies in solid tumors [14,15,16,17]. In particular, studies on animals revealed the potential activity of antibody–drug conjugates (ADC) targeting Trop-2 and established their acceptable safety profile [14]. Given the promising preclinical evidence, some of these compounds warranted further clinical development [18].

**Figure 2:**
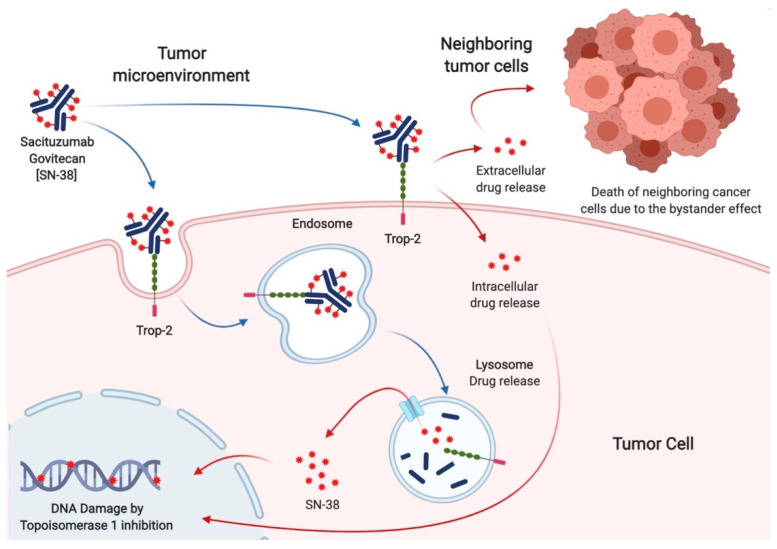
Mechanism of action of Sacituzumab govitecan. The humanized monoclonal antibody (hRS7) allows the internalization of the cytotoxic payload (SN38) into the tumor cell after binding with the surface receptor Trop-2. The hRS7 complex bound to Trop-2 is internalized through an endosomal vesicle that carries it to the lysosomes. Free SN-38, released after antibody catabolism and linker hydrolysis within the lysosome, induces DNA damage due to the inhibition of topoisomerase 1. Furthermore, the hydrolyzable linker enables SN-38 to be released into the tumor microenvironment. Given its membrane-permeable nature, free SN-38 can also exert its effect on neighboring cancer cells. Thus, tumor cells that express Trop-2 on the surface undergo the cytotoxic effect of SG by the intracellular uptake of SN38, whereas the adjacent ones experience this effect by its extracellular release (the so-called bystander effect).

Sacituzumab govitecan (SG), formerly known as IMMU-132, is a third-generation ADC specifically targeting Trop-2. It consists of an anti-Trop-2 humanized antibody (hRS7, sacituzumab) and a cytotoxic payload (SN38, govitecan) joined by a pH-dependent hydrolysable linker [17]. Unlike first-generation and second-generation ADCs, which typically contain two to four cytotoxins per antibody, hRS7 binds seven to eight molecules of SN38 [17]. Compared to older ADCs, SG is linked with a more toxic payload, effective in the range of pM instead of nM [19,20]. Indeed, SN38 is a topoisomerase I inhibitor, 100 to 1000-fold more toxic than its precursor irinotecan. The interaction between Sacituzumab and Trop-2 leads to the internalization of the cytotoxic payload into tumor cells, where it induces double-strand DNA breaks and apoptosis during the S phase of the cell cycle. Additionally, due to the acidic cancer environment, the pH-dependent linker can release SN38 from hRS7 in the tumor surroundings. This allows the killing of tumors cells lacking Trop-2 overexpression, a phenomenon known as the bystander effect, which increases SG activity by overcoming the heterogeneity of Trop-2 expression [20] (Figure 2).

Thus far, SG underwent clinical development in different tumor types, showing a remarkable efficacy in the treatment of advanced triple-negative breast cancer (TNBC). In April 2021, the drug received Food and Drug Administration (FDA) approval for the treatment of metastatic TNBC patients who failed at least two prior lines of systemic therapy [21,22]. In the same month, SG was granted accelerated approval for the treatment of advanced urothelial carcinoma, and it is currently under investigation for the treatment of many other types of tumors [17,23,24,25,26].

In this review, we provide an overview of the published and ongoing clinical trials testing SG as a single agent or in combination for the treatment of solid tumors, additionally focusing on the pharmacokinetics and toxicity spectrum of this drug.

## 2. Sacituzumab Govitecan in Breast Cancer

Sacituzumab govitecan has shown remarkable activity in BC patients since the earliest phases of its development.

The IMMU-132-01 phase I/II basket trial enrolled subjects with advanced solid tumors and set the recommended phase II dose (RP2D) of SG at 10 mg/Kg intravenously on days 1 and 8 of each 21-day cycle [21,27,28]. Of the 178 patients treated in the dose escalation and expansion parts, 53 (29.8%) had TNBC. Among them, those treated at the RP2D achieved a 31.4% objective response rate (ORR) and a 54.8% clinical benefit rate (CBR) [27]. The trial was then expanded in a selected cohort of patients, including metastatic TNBC (mTNBC) [29,30]. Preliminary results in 69 women with mTNBC who received at least one previous line of therapy showed an ORR of 30%. Final results were obtained on 108 mTNBC patients treated in the third or later line for metastatic disease. In this heavily pretreated population, SG granted a median progression-free survival (mPFS) of 5.5 months (4.1–6.3 95% CI), a median overall survival (mOS) of 13.0 months (11.2–13.7 95% CI), and a 33.3% ORR [30]. The subsequent open-label randomized phase III trial (ASCENT) compared SG with single-agent chemotherapy of physician’s choice (capecitabine, eribulin, gemcitabine, vinorelbine) in 468 mTNBC patients, pretreated with at least two lines of therapy. Median PFS, the primary endpoint, was 5.6 months in the SG arm and 1.7 months in the control arm (hazard ratio (HR) 0.41; 0.32–0.52 95% CI; *p* < 0.001), while mOS was 12.1 months with SG and 6.7 months with chemotherapy (HR 0.48; 0.38–0.59 95% CI; *p* < 0.001). In line with previous evidence, SG determined a 35% ORR, compared to 5% with standard treatment [31].

SG also showed signs of activity in patients with hormone receptors positive (HR+)/human epidermal growth factor 2 negative (HER2-) mBC patients, which represented a prespecified subpopulation in the IMMU-132-01 trial. In this study, 54 women with HR+/HER2- mBC received SG after endocrine-based therapy and at least one chemotherapy line. At a median follow-up of 11.5 months, mPFS was 5.5 months (3.6–7.6 95% CI) and mOS was 12.0 months (9.0–18.2 95% CI). Objective responses were observed in 31.5% of patients [32]. Given these encouraging results, a randomized phase III trial (TROPiCS-02, NCT03901339) is currently ongoing to compare SG with treatment of the physician’s choice (capecitabine, eribulin, gemcitabine, vinorelbine) in HR+/HER2- pretreated mBC [33].

Many other trials are ongoing to evaluate SG alone or in combination with other drugs in BC patients, both in the advanced and early settings (Table 1).

## 3. Sacituzumab Govitecan in Other Solid Tumors

Published data about the use of SG in solid tumors other than BC are still limited. However, many clinical trials are ongoing [34] (Table 2).

Solid efficacy data are available for SG in advanced urothelial carcinoma [35]. The TROPHY U-01 study is a phase II trial, which tested SG in 113 patients with advanced urothelial carcinoma progressing to prior platinum-based chemotherapy or immune checkpoint inhibitors. After a median follow-up of 9.1 months, mPFS was 5.4 months (3.5–7.2 months 95% CI), while mOS was 10.9 months (9.0–13.8 months 95% CI). Thirty-one patients (27%) achieved an objective response [25]. These results exceeded those expected in this setting on the basis of historic single-agent chemotherapy cohorts. Hence, a randomized phase III trial (TROPiCS-04, NCT04527991) is ongoing to confirm the superiority of SG over single-agent chemotherapy of the physician’s choice (paclitaxel, docetaxel, or vinflunine) in advanced pretreated urothelial carcinoma.

Preliminary signs of activity emerged also in thoracic tumors [36,37]. In the metastatic non-small cell lung cancer (NSCLC) expansion cohort of the IMMU-132-01 trial, 54 patients treated with at least one previous therapy line received SG at the doses of 8 or 10 mg/Kg. In this population, mPFS reached 5.2 months (3.2–7.1 months 95% CI), mOS 9.5 months (5.9–16.7 months 95% CI), while ORR was 19% [37]. The same trial included an advanced small cell lung cancer (SCLC) cohort (n = 50), which obtained 3.7 months (2.1–4.3 months 95% CI) mPFS, 7.5 mOS (6.2–8.8 months 95% CI), and 14% ORR [36].

Additional preclinical evidence and limited clinical experiences suggest a potential role for SG in the treatment of gynecological malignancies, including cervical and endometrial cancer [26,38,39], as well as in salivary gland tumors [23].

## 4. Pharmacokinetics and Toxicity Spectrum

The pharmacokinetic profile of SG was explored in the phase IMMU-132-01 study across patients with pretreated advanced solid tumors [27]. At the starting dose of 10 mg/kg, the peak of the antibody’s concentrations increases proportionally with continued treatment, while the half-lives of SG and free SN38 are 16 and 18 h, respectively. The clearance of systemic SG is approximately 11 to 14 h, whereas the naked antibody is cleared over about 103 to 114 h [40]. Although SN38 displays a minimal renal excretion, no data about SG administration are available for patients with creatinine clearance ≤ 30 mL/min. Similarly, the use of SG remains unexplored in patients with hepatic impairment [27]. Uridine diphosphate-glucuronosyl transferase 1A1 (UGT1A1) is the enzyme that metabolizes SN38. The activity of this enzyme can be reduced in up to 20% of the Black or African American subjects and 10% of the White subjects due to the presence of allele gene variants, such as UGT1A1*28 [41]. Subjects harboring this specific variant may present an increased risk of SG-induced toxicity, especially neutropenia, and should be closely monitored [42]. However, *UGT1A1* genotyping is not routinely recommended before starting SG [41].

Sacituzumab govitecan has a predictable and manageable toxicity profile. The most common treatment-related adverse events (TRAEs) of any grade registered in the IMMU-132-01 trial were nausea (62.6%), neutropenia (57.8%), diarrhea (56.2%), fatigue (48.3%), alopecia (40.4%), and emesis (38.6). Grade 3 or higher TRAEs occurred in 59.6% of patients. Serious AEs were febrile neutropenia (4.0%), diarrhea (2.8%), vomiting (1.4%), neutropenia (1.4%), and nausea (1.2%) [43]. One treatment-related death occurred inIMMU-132-01 trial, whereas no treatment-related deaths were observed in the ASCENT trial. In the latter study, discontinuations due to TRAEs were infrequent (4.7%), while dose reductions and interruptions occurred in 26% and 61% of patients, respectively [31].

Hematological toxicities are among the most frequent adverse events. Neutropenia represents the dose-limiting toxicity and the major cause of dose delays or reduction in clinical trials, although febrile neutropenia was observed only in about 6% of patients [43]. Therefore, primary prophylaxis with granulocyte colony-stimulating factor (G-CSF) support is not recommended [44]. However, a 25% dose reduction and the G-CSF administration is recommended in patients experiencing G4 neutropenia lasting ≥ 7 days, G febrile neutropenia, or a delay of the next scheduled dose because of G 3-4 neutropenia by 2 or 3 weeks before recovery to grade 1 [44].

Diarrhea is a class toxicity of topoisomerase inhibitors [27]. Early-onset diarrhea is caused by cholinergic hyperactivation, while late-onset diarrhea is due to the conversion of SN-38 to its inactive product SN-38G, followed by reconversion to active SN-38 by bacteria into the gut [45]. Still, the incidence of this adverse event is lower with SG compared to the historical rates reported for irinotecan monotherapy [27]. Diarrhea of any grade occurred in 62% of patients treated with SG, while G 3–4 was registered in 9% with a rate of discontinuation lower than <1%. Early-onset diarrhea should be treated with appropriate medications (i.e., atropine). In the case of late-onset diarrhea, intestinal infection should be excluded before using loperamide [44].

Sacituzumab govitecan is mildly emetogenic. Nausea and vomiting occurred up to 3 weeks after treatment initiation in 69% and 45% of patients, respectively, with G ≥ 3 rates of 6% and 5%, respectively. The prophylactic 5-hydroxytryptamine antagonist in association with dexamethasone is recommended to reduce the risk of severe toxicity and to prevent anticipatory emesis [44].

Alopecia was observed in 40.4% of patients who received SG in the IMMU-132-01 trial [43]. Although it can be the result of previous therapies in some cases, alopecia is a toxicity of SG, given the SN38 mode of action [44].

Severe dyspnea was registered in 5% of patients in the IMMU-132-01 study. This event was observed only in patients with lung metastases and did not show any correlation with interstitial lung disease [43]. Consistently, in the ASCENT trial, only one case of pneumonitis (G3) occurred [31].

## 5. Biomarkers of Response

The identification of biomarkers of response and resistance to targeted therapies is of pivotal importance in the era of personalized medicine [46,47].

The most promising biomarker of response to SG is Trop-2 overexpression. In the ASCENT trial, an immunohistochemistry score (Trop-2 H-score) based on the extension and intensity of Trop-2 membrane expression was employed to categorize the population into low, medium, and high Trop-2 expression. Median PFS among patients in the experimental arm was 6.9, 5.6, and 2.7 months for Trop-2 high, medium, and low scores, respectively. In the control arm, mPFS was numerically lower across high (2.5 months), medium (2.2 months), and low (1.6 months) H-score groups. Similarly, the ORR was 44%, 38%, and 22% in SG-treated patients with high, medium, and low Trop-2 H-scores, while it was 1%, 11%, and 6% in patients receiving standard chemotherapy [48]. Consistent results emerged by applying the Trop-2 H-score to urothelial cancer patients enrolled in the TROPHY U-01 study. In this population, ORR was 34%, 27%, and 20% among subjects with high, medium, and low scores, respectively [49]. In line with this evidence, lack of Trop-2 expression seems to confer primary resistance to SG [50].

Since topoisomerase inhibition increases double-stranded DNA break, SG could provoke synthetic lethality in patients with defective DNA repair machinery [51]. In line with this hypothesis, an in vitro study demonstrated SG efficacy in TNBC cell lines with homologous recombination repair deficiency and low/moderate Trop-2 expression [51]. However, no significant differences emerged in terms of SG activity between germline *BRCA1/2* mutated and wild-type patients in the ASCENT trial. Still, this lack of correlation may be due to the small number of *BRCA1/2*-positive subjects in both trial arms [48].

## 6. Discussion

Thanks to their unique pharmacological proprieties, third-generation ADCs are an extremely promising class of drugs on the landscape of anticancer treatment [1,42,52]. Indeed, SG represents an emerging therapeutic option for cancer patients who historically exhibit a dismal prognosis and limited treatment options, such as those presenting with TNBC and urothelial carcinomas [35,53]. Despite some criticisms regarding the conduct of the trials [54], SG efficacy in TNBC patients is consistent across all the phases of its clinical development and eventually leads to regulatory approval in this setting [30,31]. These results are particularly relevant considering they have been observed in a heavily pretreated population at a relatively low cost in terms of toxicity. A recent report confirms that patients treated with SG in the ASCENT trial experienced an improvement in health-related quality of life compared with those receiving standard chemotherapy [55]. Sacituzumab govitecan proved to be effective and is therefore approved even in pretreated urothelial carcinoma [25], but these data have yet to be confirmed by the ongoing phase III TROPiCS-04 trial. However, given the pleiotropic nature of Trop-2, it is possible to hypothesize that SG could find an indication in a broader set of diseases, as suggested by the increasing number of early phase trials in tumors other than breast or urothelial carcinoma.

As evidence about SG efficacy accumulates, the need to identify and overcome potential mechanisms of resistance to this drug grows stronger. To date, alterations involving the genes *TACSTD2* and *TOP1*, codifying for Trop-2 and topoisomerase-1, respectively, seem to be involved in acquired resistance to SG [50]. Despite its clear efficacy, a potential limitation of SG use is represented by its cost. According to a recent analysis conducted in the United States and China, the price of SG should be at least halved to achieve cost-effectiveness [56]. This practical consideration poses relevant challenges in terms of drug’s accessibility, especially for those patients living in low-income countries.

In the foreseeable future, specific strategies could be tailored to overcome SG resistance. For example, patients harboring a *TCSTD2* alteration could be treated with a different ADC incorporating the same payload, whereas subjects displaying a *TOP1* mutation could still benefit from anti-Trop-2 directed therapies [50]. Additionally, multi-drug regimens could enhance SG activity and delay the onset of resistance [57,58,59,60,61]. Among those, the combination of SG with PARP-inhibitors has a strong preclinical rationale, and the preliminary evidence of clinical activity is still available [57,58]. Antibody–drug conjugates could also exhibit a synergistic effect with immune checkpoint inhibitors, and trials combining SG with immunotherapeutic are ongoing [59,60].

In conclusion, SG has already entered clinical practice due to its solid efficacy and safety results in treating specific tumor types. However, further preclinical and clinical studies are needed to fully understand and exploit the therapeutic potential of this ADC.

## Figures and Tables

**Table 1:** Selected published and ongoing trials testing Sacituzumab govitecan in breast cancer.

Trial Name	Phase	Study Treatment	Study Population (Number Enrolled If Available)	Study Design	Status (Ref. If Published)
NCT01631552	I/II	SG	Solid Tumors (515)	Open label, single group	Published [30,32]
NCT04039230	I/II	SG + Talazoparib	mBC	Open label, single group	Ongoing
NCT03424005	Ib/II	SG + Atezolizumab	mTNBC	Open label, randomized multi-cohort	Ongoing
NCT03992131 (SEASTAR)	Ib/II	SG + Rucaparib	Advanced solid tumors	Open label, non-randomized	Ongoing
NCT04927884	Ib/II	SG	mTNBC	Open label, single group	Ongoing
NCT04647916	II	SG	HER2-BC and brain metastases	Open label, single group	Ongoing
NCT04468061 (Saci-IO)	2	SG + Pembrolizumab vs. Pembrolizumab	mTNBC	Open label, randomized	Ongoing
NCT04448886 (Saci-IO HR+)	2	SG + Pembrolizumab vs. Pembrolizumab	HR+/HER2-mBC	Open label, randomized	Ongoing
NCT04230109 (NeoSTAR)	2	SG + Pembrolizumab vs. Pembrolizumab	Localized TNBC	Open label, randomized	Ongoing
NCT02574455 (ASCENT)	3	SG vs. Chemotherapy	mTNBC (529)	Open label, randomized	Published [31,32]
NCT03901339 (TROPiCS-02)	3	SG vs. Chemotherapy	HR + HER2-mBC	Open label, randomized	Ongoing
NCT04595565 (SASCIA)	3	SG vs. Chemotherapy	HER2-/BC wo pCR after NACT	Open label, randomized	Ongoing

Legend: BC: breast cancer; HER2-: HER2 negative; HR+: hormone receptor positive; m: metastatic; NACT: Neoadjuvant therapy; pCR: pathologic complete response; SG: Sacituzumab govitecan; TNBC: triple-negative breast cancer; wo: without.

**Table 2:** Selected published and ongoing trials testing Sacituzumab govitecan in solid tumors other than breast cancer.

Table	Phase	Study Treatment	Study Population (Number Enrolled If Available)	Study Design	Status (Ref. If Published)
NCT04617522	1	SG	Advanced solid tumors and moderate liver impairment	Open label, non-randomized	Ongoing
NCT04724018	1	SG + Enfortumab vedotin	mUC after platinum and anti-PD1/L1	Open label, single group	Ongoing
NCT03995706	1	SG	Breast cancer with brain metastases and glioblastoma	Open label, single group	Ongoing
NCT01631552 (IMMU-132-01)	1/2	SG	Epithelial cancers	Open label, non-randomized	Published [27]
NCT04826341	1/2	SG + Berzosertib	Advanced solid tumors > 1 L SCLC after platinum, HRD cancers after PARPi	Open label, non-randomized	Ongoing
NCT04863885	1/2	SG + IPI/NIVO	1L cisplatin-ineligible mUC	Open label, non-randomized	Ongoing
NCT03869190 (MORPHEUS-UC)	1/2	SG + Atezolizumab	mUC	Open label, randomized, multi-cohort	Ongoing
NCT03992131 (SEASTAR)	1b/2	SG + Rucaparib	Advanced or metastatic solid tumors	Open label, non-randomized	Ongoing
NCT03337698 (MORPHEUS-Lung)	1b/2	SG + Atezolizumab	mNSCLC	Open label, randomized	Ongoing
NCT03547973 (TROPHY U-01)	2	SG	mUC after platinum or anti-PD1/L1 (113)	Open label, non-randomized	Published [25]
NCT04251416	2	SG	Persistent/recurrent EC	Open label, single group assignment	Ongoing
NCT03964727 (TROPiCS-03)	2	SG	Metastatic solid tumors	Open label, single group assignment	Ongoing
NCT04559230	2	SG	Recurrent glioblastoma	Open label, single group assignment	Ongoing
NCT03725761	2	SG	Castration-resistant prostate cancer after second-generation ADT	Open label, single group assignment	Ongoing
NCT04527991 (TROPiCS-04)	3	SG vs. TAX/TXT/Vinflunine	Metastatic or locally advanced unresectable UC	Open label, randomized	Ongoing

Legend: 1/2L: first or second line; ADT: androgen deprivation therapy; HRD: homologous repair deficiency; IPI/NIVO: ipilimumab + nivolumab; m: metastatic; EC: endometrial cancer; NSCLC: non-small cell lung cancer; PARPi: poly (ADP-ribose) polymerase-inhibitors; SCLC: small cell lung cancer; SG: Sacituzumab govitecan; TAX: paclitaxel; TXT: docetaxel; UC: urothelial carcinoma.
